# Book Reviews

**Published:** 1861-10

**Authors:** 


### OUR LIBRARY TABLE.

*The Roll of the Royal College of Physicians of London;* Compiled from the Annals of the College and from other Authentic Sources. By William Munk, M.D., Fellow of the College, &c. Vol. I. 1518—1700. 8vo. Longman.

This is a very valuable addition to what may be termed Class-biographies, and supplies a want in the history of science in England. The industrious author, Dr. Munk, being sensible of that *desideratum,* had deposited his carefully collected materials in the library of the College of Physicians for the use of his brother Fellows, without any view to their being published. But the Council, justly appreciating their merits, and aware of the benefit which others than the members of the corporation would be likely to derive from such a work of reference, ordered the Roll to be printed. Accordingly, while thus paying a proper tribute of respect to Dr. Munk, they have entitled themselves to the thanks as well of the profession as of those who take an interest in the lives of the learned. For the names of more than two-thirds of the individuals commemorated in the volume, we should search in vain the columns of our most extensive biographical dictionaries.

While endeavouring to record as much as is essential to the history of those early physicians, Dr. Munk has with proper judgment abstained from all irrelevant matter or parade of authorities, with which a less judicious compiler would have overlaid his work. He has had to travel in many obscure by-paths of the past, and where research has proved unsuccessful, the failure has evidently proceeded from no want of pains. As the Roll will take its place hereafter with the *Athenæ* of Wood and of the Messrs. Cooper, and similar standard authorities, we note a few points that have occurred to us in perusing it, which may be serviceable in the event of a second edition.

The Dr. John Craige, whose memoir occurs at p. 112, was the third son of the eminent lawyer Sir Thomas Craige, of Riccarton, whose treatise *De Feudis* is one of the noblest monuments of the legal literature of Scotland. That he was a friend of the philosopher of Murchison there is no doubt, but that Napier was indebted to him for the idea which led to the discovery of logarithms has been disproved by Mr. Mark Napier, in his elaborate life of that illustrious inventor, where particulars of Craige's intimacy with Tycho Brahe may be seen.

In regard to Sir Francis Prujear, the celebrated President of the College of Physicians, we should have liked Dr. Munk's authority for assigning Essex as his native county. That he possessed considerable estates there is well known, but we have some doubts as to his having been born within its confines. His first wife was of the old family of Legat, of Dagenham. A portrait of him is in the possession of the last representative of his family, Mr. Francis Prujear, barrister-at-law, to whom also at one time belonged a portrait of his son, Dr. Thomas Prujear, which has been unfortunately lost.

It seems to have escaped Dr. Munk, while sketching his memoir of Sir Theodore Mayerne, that some twenty volumes in autograph of that distinguished physician exist among the Sloane MSS. in the British Museum. One of these contains memoranda of his professional attendance on King James I.; the others, entitled *Ephemerides*, form a journal of all the cases which he attended from 1603 to 1649. These are especially curious, from the very minute statements which they give, not only of the symptoms of the disease, and the remedies prescribed, but of the personal formation, state of the organs, peculiarities of diet, affections and antipathies of the patient, as also of the complaints to which his parents had been liable. As Mayerne was the court physician of his age, the vast majority of the cases were those of the aristocracy of France and of Britain. They are all written in Latin, and, if printed with suitable annotations, would form the most interesting medical annals ever published. Can the Council of the College be induced to bestow another favour on the public by producing these relics of so noted a member of their body, under the editorial care of Dr. Munk, whose capabilities for such a task are so well exhibited in this Roll? A reprint of the Case-book of Dr. Thomas Hall, the son-in-law of Shakspeare, would also be acceptable.

*A Treatise on the Surgical Diseases of the Eye.* By HAYNES

Walton, Surgeon to the Central London Ophthalmic Hospital, and to St. Mary's Hospital. Second Edition. Re-written. 173 Illustrations. 8vo.

Among the notable surgical books of the year, is a second edition of this work, and it is certainly what the preface expresses, "a new edition in the fullest sense of the word." With several new chapters, and many re-written, some re-cast, and all improved, this is, indeed, a new book which contains all that is good and trustworthy in ophthalmic surgery up to the present hour, issuing from a highly accomplished and successful surgeon in large practice, written with remarkable clearness, well printed, and most beautifully illustrated.

While for a long period the great advantages of chloroform in general surgery have been recognised, it is only now that we have its application to operations on the eye well defined. In a chapter which is sufficiently full in all that relates to this subject, including important rules for administering the drug, we learn when the chloroform is advantageous, when it may or may not be given according to the patient's desire, and when it is profitable to withhold it.

Injuries and accidents to the eye are practically and scientifically discussed. Besides the individual personal importance attaching to these, there has of late arisen the legal question of compensation, and in Mr. Haynes Walton's volume will be found the solution of many problems heretofore not understood. How it is that vision may be lost from a blow on the eye so slight as to make no external mark, or a stroke on the forehead, or head, is here shown. The causes are demonstrable, the physical changes are palpable by the aid of the wonderful ophthalmoscope.

Following the preceding subject is that of sympathetic implication of the eye. Our author shows that the general opinion respecting this is erroneous, and that any injurious sympathetic action almost exclusively arises out of wounds, or injuries that disorganize an eye.

The many delicate operations on the eyeball are described in a masterly manner, and the surgical treatment of squint, strange to tell, only till lately perfected, is well explained, and, according to what we are here told, reduced to certainty, if there be a proper selection of cases; no difficult matter, it would seem.

A special chapter is devoted to iridectomy and other operations for the cure of glaucoma. The following remarks on the former operation will be read with interest:—

"That the operation has been recklessly performed by being applied to cases to which it is wholly inapplicable, according even to its propounder's theory, I have had frequent proof. It is useless to give particulars. Graefe, as I must remind my reader, lays great stress on the curative results, according to the stages of the disease.

have had the satisfaction in ascertaining, by actual inspection, that some of these—all that I have been able to watch—have completely recovered.

"But all these things tell nothing against iridectomy, if it be a valuable operation. Certainly not; they only show the abuse of the measure. The same indiscretions may, and do, attend our most valuable operations. A man may operate for stone where there is none in the bladder; or proceed to relieve a strangulated hernia, where there is no rupture. But in the present instance, with the existence of great difference of opinion about the utility of the procedure, and when men are seeking for evidence from facts, the exposure of such error and malpractice is of value, because it shows that there may be conclusions from very insufficient, as well as wrong data.

"It remains for me to give the result of my experience from what I have seen of the operation in glaucomatous eyes, in the practice of others, and from my own. Respecting the first, the cases have been, with but few exceptions, of the chronic form of the disease. In some of them there was certainly a slight improvement of vision for a few days, but in none has this been more than temporary. Pain has also been relieved, and, in a few, has been so long absent, that it has been supposed to be for ever removed, when, with sad disappointment, it has returned. Some of these had been published as most successful cases. Of the acute kind I can give no better report.

"My own operations have not been numerous. Having been disappointed in the result in some well-selected cases of acute glaucoma (and these have been few, for with no small field of observation I do not find such cases common), I could not make that strong recommendation to patients which they required respecting the success of an operation, or the possibility of success, to induce all to submit to it.

"While I wish it most fully to be understood that I do not condemn iridectomy, I must express my own conviction that I attribute all the good effect which may follow to the mere tapping of the aqueous humour. I have found as much benefit to sight and reduction of pain from this, as I have been able to trace to the other measure. In a private patient of mine, seen by several other medical men, there was sub-acute glaucoma in one eye of nine months' standing. This lady had coruscations, and much pain. She could read nothing. The iris bulged, and the pupil was slightly dilated. Two days after the first tapping she could manage to read a part of one of the articles in the *Times*. The pain was relieved, and the coruscations lessened. Vision then got as bad as before, and a slight attack of acute inflammation supervened. Several tappings, at intervals of a week, enabled her to read large type, but not with clear vision, The pain quite left her, and the coruscations almost disappeared. This improved state lasted five months, as long as I attended her; during which time there was no accession of inflammation. The vitreous humour was always too hazy to afford a satisfactory examination of the fundus of the eye.

"I do not by any means infer, or wish it to be understood, that I believe tapping the aqueous humour to be a remedy for glaucoma. That it is capable, occasionally, of affording relief, I am sure; but as I have not yet practised it, either as extensively as I wish, nor with that degree of accurate observation which is necessary to establish facts, I would rather say no more about it.

"There is quite authority enough, even from some of our English surgeons, to warrant any inquiring student to undertake iridectomy. If a man, whose opinion in surgical matters is considered to be of a superior kind, more especially in ophthalmic subjects, speaks of it as a sure and certain remedy in glaucoma, and as arresting the disease and restoring sight in a marvellous manner, surely it ought not to be left untried. I will merely suggest that, if the trial be made, fitting cases should be selected; and that full and well-authenticated reports, extending over a sufficient period, be given by the operator to the public; and withal, that the facts be attested by others. The anonymous reports in the medical journals, on most operations, are, as a rule, of less value, when accuracy is needed, than is supposed; and in the present case, those that have been published are not exempt from this charge."

Mr. Walton's work concludes with a chapter on the ophthalmoscope, the great discovery of the age in this department of medicine, and which owes its origin to an English medical student, although undoubtedly the Germans perfected the discovery and made it available for practice. With this instrument, or speculum, with which we can explore the interior of the eye, diseased states, of which formerly no knowledge existed, are clearly seen and understood as if they occurred on the surface of the body. The earliest stage of cataract, heretofore inscrutable, can be most readily and quickly discerned. Indeed, the student of the present day, with the ophthalmoscope, is able to understand more about the diseases of the eye than the veteran professor of a past generation. Mr. Walton's treatise contains all about the subject in a practical form.

*De l'Etude de la Folie.* Par Dr. J. M. Guardia. Paris: 1861. pp. 32.

Dr. Guardia announces that the higher studies languish and die in France, that though the educational machine appears to move pleasantly and prosperously, its products are inadequate to justify veneration for the system pursued, and that nothing foreshadows a return of energy and usefulness. From this sweeping censure Claude Bernard and Berthelot are excepted, as fulfilling the high functions of sages and teachers. At the root of this decadence are placed the exclusion of physiology from the domain of metaphysics and theology, the puerile attachment to the arbitrary dualism, or distinctions between spirit and matter, and the panic inspired by materialism. A review of the works devoted to the exposition of mental disease which have appeared during the last sixty years will demonstrate how and where the true and fruitful investigation of the subject has been receded from or approached, and may trace these results to the individual characteristics of the inquirers. In the opinion of Stahl, the soul was the principle and directing influence of life, the guide and governor of the organization; it suffered from the disorders and rebellion of the viscera as a just punishment of original sin—it was conquered and humbled in the struggle maintained in order to master its slave—from the body came its imperfections and sorrows. His spiritualism or platonism tempted him to evade the difficult problem as to the seat of the mental diseases, and he preferred the elucidation of such nervous affections as hysteria, hypochondriasis, &c., the origin of which he referred to the abdominal organs. He was the last of the ancients. After the liver, heart, stomach, diaphragm, had each in turn occupied and engrossed attention, and after the brain was reduced to its old hyppocratic and servile position of being a gland to expurgate the humours, the works of the bold innovator, Borden—himself an admirer of antiquity, but the precursor of the modern school of medicine—gave to the nervous system that preponderance in the animal economy which has since been universally admitted. This law was proclaimed coincidently with the period when reason assumed its real rank and sway among the human faculties. In this grand, it may be designated the encyclopædic, era of progress in France, appeared the observations of Fouquet—a disciple of Borden—on insensibility, Bichat, Cabanis, and Vicq-d'Azyr followed, leaving valuable researches upon the brain and nervous centres. As a discoverer of great and original mind, as an intrepid philosopher, and as an accurate and consistent physiologist, the striking merits and services of Gall must be acknowledged by every man. Although unrewarded and uncanonized, he has this distinction, of having promulgated a thorough reform in our conceptions of the higher mental powers.

Like the knowledge of the nervous system, the study of mental disease is modern. The priests burnt the insane as possessed, the philosophers arrived at no more correct views of the derangement of intellect than the maniacs themselves. Friederich shows how numerous and how illusory were the attempts to grapple with this class of affections until an intimate knowledge of the phenomena of the nervous system was attained. To Pinel alone belongs the glory of first carrying into the study and treatment of insanity clear views and humane sentiments. He belonged, however, to the eighteenth century—he was timid, undecided, and the slave of scholastic influences. At once a man of progress and a man of routine, an innovator and original, but vague and unsteady, he gave the signal for battle even before Bichat; he recalcitrated, then re-entered the strife, and ultimately fell into and disappeared along the backward groove. Esquirol, his worthy successor, an observer, in the strict sense of the word, possessed those second-rate qualities which constitute a physician. His sagacity did not penetrate beyond the fact—he saw, noted, and described faithfully phenomena, without seizing the relations which give them significance, not perceiving, perhaps, the necessity of working out the subject synthetically. He had the tendency of narrow but practical intellects of doubting that generalization is compatible with practice.

His works are a collection of memoirs, a repertory abounding in facts, but they do not develope a law or a theory. Unlike his predecessor, he was absolute, exclusive, confined himself to his speciality, and repudiated alike the authority of his master, and the innovations which pressed upon him from all sides and sources. The capital and fatal errors of these great minds were, that they were reactionary in the revolution in science which swept around and past them; and that they were hostile to the views of their contemporaries, Gall and Broussais. These regenerators have lived in anticipation of a more developed state of intelligence, capable of appreciating and applying the solemn and essential truths which they advocated. Their creed may be summed up in the aphorism, there can be no function without an organ, no psychical phenomenon without a nerve. Georget, Lallemand, Leuret, are enumerated as eminent disciples of this school; but although Dr. Guardia affirms that the design of his essay has been to show that the principle of life has been misunderstood, that it is necessary to go back to a cerebral physiology which shall not, like the psychology of the present day, be confined to the intellectual faculties, but which shall include the instincts and propensities and sentiments demonstrated to be connected with the base and posterior parts of the brain; one object is constantly before him, he offers homage to another and a most distinguished member of this group. The pamphlet is as much an eloge of M. Falret, senior, as a monody upon the state of medical education. Besides incidental tributes, there is to be found at pp. 11, 17, 26, a laboured panegyric upon this physician, who may be justly described as not merely the representative of a particular dogma or doctrine, but as at the head of the Psychological School in France. So entirely do we coincide in the description given, that we shall quote one of many similar passages:—

"He has found in the study of mental diseases a compensation for the sacrifices entailed by the choice of a liberal career, and of obeying a particular mission. In his difficult speciality, he stands alone and eminent. This lofty position has been secured by his rare sagacity, a profound knowledge of the human understanding, a wide experience; a rational and successful mode of treatment; a suggestive and effective style of teaching; profound writings, characterized by sound sense, original and vigorous expositions, minute and refined criticism, and progressive tendencies indicative of the originality and comprehensiveness of his mind."

Accepting his observations in the light of a somewhat special pleading in favour of a certain school and opinions, it does not seem necessary, after the analysis introduced, to take exception to the bold, it is suspected rash, style of the advocacy; nor to the onesided representation which he has given of the opinions themselves, and of the relations which they hold to the physiological views, or the actual practice of those by whom they are entertained. We are unworthy co-believers; but our convictions have ever been, that to treat mental disease solely in reference to the known connexion between mind and nervous matter, would be absurd and fruitless; equivalent to confining the attention in chorea to the muscular system; and that the true pathology was to regard insanity as a symptom of various diseases, situate in solids or fluids as the case may be, expressed and exhibited through the nervous system; and that the legitimate and successful treatment must be directed to the removal or mitigation of those structural changes, it may be in the digestive organs or in a nerve cell, upon which delirium, or depression, or perverted affection depend. That erroneous views or imperfect knowledge, as to this subject, prevail, or are likely to prevail, among the members of the medical profession is rendered probable, if Dr. Guardia's statement "that instruction in psychology is reduced to a few lectures, delivered once a week, for three months, by Baillarger, the zealous physician of Salptérière," be received without qualification. The abrupt declaration, "clinical teaching does not exist," sets at nought the hundreds of youths now engaged as medical assistants in public, departmental, and private asylums throughout France, who enjoy the most extensive means of observation and instruction, and in what is correctly styled the clinical form. The results of this system, the exclusive and contracted character of which is to be lamented, may be seen in the assembly of great and gifted men who now constitute the Société Medico-Psychologique.

*Lectures on the Diseases of the Kidney, generally known as "Bright's Disease," and Dropsy.* By S. J. GOODFELLOW, M.D. Lond. F.R.C.P., Physician to the Middlesex Hospital, &c. London. 1861. 8vo, pp. 306.

This is one of those thoroughly useful books which are at all times a boon to the actively engaged practitioner. It is the work of an accomplished and thoughtful physician; and together with the results of his own practice and observation, presents an excellent and valuable summary of all that is known upon the subjects of which he writes. The lectures were originally published at intervals in the *Medical Times and Gazette;* but the author has acted wisely in gathering them together in one volume. The mode in which Dr. Goodfellow treats his subjects will be best shown by an example. The following, taken almost at random, refers to the pathological action of alcohol:—

"That alcohol is a local irritant is unquestionable, and that it produces its effects upon the system partly in this way is very probable. It may act remotely by sympathy to some small extent, as Orfila believed. But we have seen from the very able researches of MM. Lallemand, Perrin, and Duroy, from whose book I have already quoted so largely, that it is rapidly absorbed by the venous radicles, and that its principal action is directly upon the different organs which it irritates, and eventually inflames. Especially has it been proved to be present in greater proportion in the nervous tissue than elsewhere, which it more particularly excites. It disturbs its functions; it perverts and ultimately destroys the intellectual, and even the emotional faculties; it disturbs the function of the sensory nerves, both common and special, as shown by subjective tactile phenomena, strange perversions of taste, double vision, and other disorders of the optic nerves, tinnitus aurium, and other disorders of the auditory nerves. It equally disorders and destroys the functions of the motor nerves, as shown in irregularity and absence of consentaneous action of the movements. From these effects upon the cerebro-spinal system, it is more than probable that it disturbs and impairs the functions of the organic nervous system, as evidenced by defective nutrition and secretion. When taken in the form of brandy, whisky, gin, and such fluids, it impairs nutrition, probably from its great attraction for water, inspissating the blood and juices of the body. I need not mention in what large proportion water enters into the composition of the tissues and fluids of the body. It is probably in this way that it acts as a diuretic, so far as the increase of the watery part of the urine is concerned, not only from the increased quantity of water ingested with and after the brandy, but from its abstracting it from the tissues. There is no doubt that it tends to harden the brain substance, and to produce atrophy of many of the structures, not only by increasing the quantity of connective and other white fibrous tissues, and so leading to undue pressure upon the more important parts, but by condensing the fissures directly by the abstraction of water. There is no doubt of its exerting this destroying influence upon the liver. I shall endeavour to show you that it does so upon the kidney also. As a general rule, it irritates and inflames the tissues of the stomach and duodenum, and even the pancreatic and hepatic ducts, and it probably affects and deteriorates the secretion of these glands. It produces hypertrophy of the connective tissue forming Glisson's capsule, which, in its turn, presses upon the small vessels, and upon the hepatic cells, and produces atrophy of these anatomical elements in two ways, first, by cutting off the supply of nutrient materials; and, secondly, by absorption from pressure. It probably exerts a direct effect upon these cells, leading to their destruction, independently of those produced by the thickening of Glisson's capsule. The digestive processes are probably still more impaired by the bad quality of the bile and pancreatic secretion.

"Now, very much the same changes take place in the kidney as in the liver and other organs. We have seen that alcohol passes through the vessels and tissues of this organ as alcohol; it irritates these tissues, as it does similar tissues in other parts; it leads to blood delay; it impairs the influence and function of the nervous system; it produces hypertrophy of the connective tissues, forming the stroma or framework of the organ and of the capsule; and it produces a granular appearance precisely as it does in the liver. In fact, this alteration is very commonly seen in both these organs in old drunkards, especially and almost exclusively those who take the raw spirit in large quantities, or spirit mixed with only small quantities of water. Those who drink largely of beer, and perhaps of wine, are found to have a somewhat different form of kidney from those who drink it in other forms, especially when taken as gin, brandy, &c. But we have seen that alcohol separates and modifies the fatty matters of the blood. MM. Lallemand, Perrin, and Duroy, have seen this. Most pathologists believed that so far as the relation between cause and effect could be traced, it was almost certain that alcoholic beverages, when largely and continuously consumed for any length of time, led to fatty degradation. This separation and alteration of the fatty principles of the blood, have now been actually seen and proved, and probably play a very important part in the pathological effect of alcohol, when taken in large quantities, in the form of brandy, gin, whisky, &c. Saponifiable fatty matters that are visible to the naked eye are calculated to impede the circulation through the capillaries—if not to cut off the blood-supply altogether—and so produce atrophy of the secreting tissues, while the connective tissues, supporting the vessels, would receive an undue supply of blood plasma, and therefore become hypertrophied. It is not improbable that some of these fatty matters become transuded with the exudates, and thus lead to the presence of fat in the tubular, and also in the inter-tubular, substance; some may also remain in the walls of the capillary vessels, and replace in time the normal elements."—(pp. 187—91.)

*Meteorology.* By Sir JOHN F. W. HERSCHEL, Bart., K.H., M.A., D.C.L., F.R.S. L. & E., &c. Sm. 8vo, pp. 288.—*Physical Geography.* By the same Author. 8vo, pp. 441. Edinburgh: A. and C. Black. 1861.

These two works are reprints of the articles on these subjects in the last edition of that great literary and publishing achievement, the *Encyclopædia Britannica.* The present publications have been revised and certain additions made to them by the distinguished author; and in their present form they furnish what has long been wanted, handy, inexpensive, yet withal beautifully printed, and thoroughly trustworthy manuals of the subjects on which they treat. It is on this account that we are particularly desirous to direct the attention of students and lecturers to these two works—the one the necessary complement of the other.

To the scientific medical man a competent knowledge of meteorology and physical geography is simply essential. Without it not only several of the chief problems in biology and the causation of disease are inextricable mysteries, but even the ordinary phenomena connected with the origin and development of maladies can be but imperfectly comprehended. Meteorological and physical-geographical facts intrude themselves largely, both into the medical and physiological, and the former have a distinct place in the chemical, courses of our Schools of Medicine. The weather, the seasons, and climate, the peculiar subjects of meteorology, have special and all-important medical aspects, and neither the weather, nor seasons, nor climate, can be rightly understood apart from physical geography.

Judging from our own experience, both students and teachers often feel the want of a text-book on both subjects, which while sufficiently brief not to exact too much from the time of the former, could be recommended without hesitation by the latter. This want is admirably supplied by the publication of Sir John F. W. Herschel's essays in a separate form.

*The Platonic Dialogues for English Readers.* By WILLIAM WHEWELL, D.D. Vols. II. and III. Cambridge: Macmillan. 1860-61.

Dr. Whewell has achieved the great task of bringing Plato within the reach of every English reader. When the first volume of the *Platonic Dialogues* appeared, we directed the attention of our readers at some length* to the advantages arising from the publication, and the gratitude due to the Master of Trinity, from all readers to whom the original was a shut book, for the happy idea and admirable execution of the work. We have now to record the publication of two additional volumes. The second volume contains what Dr. W. terms "the Anti-sophist Dialogues," "inasmuch," he writes, "as they are mainly occupied with discussions in which persons who have been called 'sophists' by Plato and by his commentators, are represented as refuted, perplexed, or silenced. Of such persons there will be found in the following pages, Protagoras, Prodicus, Hippias, Gorgias, Polus, Callicles, Ion, Euthydemus, Dionysiodorus, and Thrasymachus, who is, however, much more prominent in the First Book of the Republic. But though these persons are all included by some of Plato's admirers under the term *sophists*, are all involved by many commentators in that charge of false reasoning and sinister purpose which *we* imply by the term, and are looked upon by many persons as a sect or party who made common cause, corrupted the moral principles of the Athenians, and were unmasked and put down by Plato; they were, in truth, most diverse in their tenets, characters, position, mode of discussion, and objects; and were, several of these, as strenuous inculcators of virtue and as subtle reasoners as Plato himself." The dialogues contained in this volume are, Protagoras, the Greater Hippias, the Lesser Hippias, Ion, Euthydemus, Gorgias, Phædrus, Menexenus, and Philebus.

The third volume contains the Republic and the Timæus. In the preface of this volume Dr. Whewell writes:—

"I cannot but believe that the English reader, though he may sometimes be

---

* *Journal of Psychological Medicine*, vol. xiii. p. 144.

disappointed with the results of Plato's speculations, will find, in that portion of the Platonic Dialogues which I have now completed, a very striking body of writings. It appears to me also, that these writings become more striking by being taken in the order in which I have presented them. The points discussed in the *Laches*, the *Charmides*, the *Lysis*, the *Rivals*, the *Alcibiades*, though involving weighty questions, are in a great degree juvenile puzzles, belonging to an early stage of Moral Philosophy. After these, the fine dramatic delineations of other moral teachers and disputants, Protagoras, Prodicus, Hippias, Gorgias, Polus, Ion, Thrasymachus, form an extraordinary gallery of philosophical portraits. And this depiction is further graced by a lofty tone of virtuous resolve, as in the *Gorgias*, and by a thorough enjoyment of literary beauty and literary playfulness, as in the *Phædrus*; while through all there runs a stedfast assertion of the great doctrine of the Immortality of the soul, presented as the belief of Socrates in the great tragedy of his death, the *Phædo*, and again urged in various mythological forms in the *Gorgias*, the *Phædrus*, and the *Republic*; add to this subtle speculations concerning the soul and its faculties, anticipating the most acute analyses of modern psychologists:—and we have, I think, matter in which the English reader may find ground for an admiration of Plato, and a pleasure in reading him, not altogether disproportionate to the reputation which belongs to his name.

"That Plato's arguments are sometimes inconclusive, sometimes unfair, and his dramatic representations of opponents sometimes caricatures, are criticisms to which he has been subjected from his own day to ours; and the justice of them will not be denied, I think, by any one who undertakes to make sense of what he has written. I am aware that there have been persons who have explained all seeming inconsistencies and weaknesses in him by ascribing to him a habit of writing ironically. To suppose that Plato is an author whose habit is to lay traps for unwary readers by saying the opposite to what he means, would be to make him the dullest of jesters; and I should hope there are few of his Greek readers who have so poor an opinion of him.

"The *ethical* system of Plato is completed in the Dialogues which I have now published. There are other Dialogues of great interest, as the *Banquet*, the *Cratylus*, the *Theatetus*, which I have not yet translated. Whether I shall venture to undertake these, circumstances must determine."

We trust that these circumstances may prove favourable.

*Medical Jurisprudence.* By ALFRED JEROME TAYLOR, M.D., F.R.S., F.R.C.S., &c. Seventh edition. London: 1861. 8vo, pp. 947.

If the publication of a seventh edition of this important standard work does not convey to the reader a sufficiently clear idea of the degree of estimation in which it is held, let him learn from the preface, that since its first publication in 1844, fifteen thousand seven hundred and fifty copies have issued from the press. In the present edition the author states that he has not found it necessary to make any extensive changes, but some articles have been abridged in order to make room for more recent information, and every chapter has been carefully revised throughout so as to place the work on a level with the progress of medical and legal knowledge.

*Health and Disease as influenced by the Daily, Seasonal, and other Cyclical Changes in the Human System.* By EDWARD SMITH, M.D., LL.B., F.R.S., Assistant Physician to the Hospital of Consumption and Diseases of the Chest, Brompton, &c. London: 1861. 8vo. pp. 399.

This is a work of singular interest and importance. The writer justly says that "no work of modern date exists in which the cyclical changes proceeding in the human system are described, or in which even the influence of season is cited at any length to the causation and treatment of disease." He seeks to supply the want here indicated and to place our knowledge in this respect upon a more exact and scientific basis. To effect this he has submitted himself and others to a series of experimental observations, extending almost without intermission over a period of six years. The results of these observations have been presented from time to time before various learned societies, but in the present work the whole of the author's researches are for the first time embodied, and their application to health and disease set forth, in a connected form. From the late period at which we have received Dr. Smith's work, we are, in justice both to the author and his subject, reluctantly compelled to postpone its consideration until our next number.

*Brief Notes of a Visit to the Leper Hospital, at Granada.* By John Webster, M.D., F.R.S., &c.—pp. 13.

In this highly interesting pamphlet, reprinted from volume XLIII. of the *Medico-Chirurgical Transactions*, recently published, Dr. Webster succinctly describes various observations made by him when inspecting, during a previous autumn, the ancient institution he has named, it being one of the oldest charities throughout Europe, and originally founded by Isabella, Queen of Spain, more than three centures and a half ago; that is, soon after Granada was wrested from Moorish domination.

Believing some notice of the salient features which leprosy presents, as also a summary of the facts detailed, may prove interesting, we would here observe, that when visited by Dr. Webster in September, 1859, the establishment contained 53 inmates; 39 being male, and 14 female patients; showing that the former sex greatly predominated in number over the latter. This excess of male lepers has always prevailed, and experience proves that men are more frequently affected than women, in the proportion of about five to two at the least; while, in reference to age, among these 53 leprous sufferers there were individuals varying from a girl fourteen years old, and a lad not much more advanced, to persons of both sexes verging towards their grand climacteric. Again, as to the complaint itself, some patients appeared only recent victims, whereas others had been several years attacked; while all seemed slowly but surely advancing to a fatal termination.

Various cases have the mouth and tongue ulcerated, sending forth a horrible stench; their voice then becoming weak and husky, so that they could only speak in a whisper; while some were without an eye, or part of their face was eat away by extensive sores. Several had lost fingers, toes, and even a hand, in consequence of ulceration; whereby the poor sufferer became a horrid spectacle of mutilation and helplessness. Two or three were all but bedridden from their physical sufferings or debility; and two formed such a mass of bodily corruption, that it would be difficult to give any description.

One particular phase of the mental faculties noticed in leprous victims deserves notice—namely, *they almost universally seemed happy, and even quite contented with their really sad condition.*

Another curious characteristic likewise merits record,—namely, numbers were very lascivious in conversation, and their conduct would have become equally objectionable, but for the strict surveillance constantly exercised to keep the sexes separate, since in both the "*libido inexplebilis*" of ancient authors often constitutes a prominent symptom among such parties.

Regarding the particular districts from whence lepers at the Granada Hospital usually came, it may be stated that very rarely any were natives either of the city or adjacent "Vega," which is one of the most fertile regions throughout Spain. Almost all the patients being from places adjoining the low, south-eastern Mediterranean sea-shore, especially Almeria, Adra, Motril, Malaga, Velez-Malaga, or adjacent villages—Cadiz and its vicinity being also affected. Hence, the malady would seem as if especially obnoxious to marine populations, not persons who dwell habitually upon more elevated or inland localities.

Although leprosy is now very rarely seen in countries where formerly it proved exceedingly common, modern Spanish observers assert confidently that the malady has been lately increasing in number throughout various situations of the Peninsula; more victims of the disease being at present recognised than during the early part of the current century.

That the complaint still prevails to a considerable extent in some regions of Spain, appears to be conclusively illustrated by an instructive fact recently communicated to the Madrid Royal Academy of Medicine, by Senor Mendez Alveiro—namely, that 284 leprous patients were ascertained to be alive during 1851 in nine Spanish provinces, without reckoning many more about whom no statistical return had been procured, at that date, from the districts where they resided.

With reference to the causes chiefly influential in producing leprosy, although hereditary tendency plays, according to some writers, a considerable part, the author thinks such an opinion very doubtful if not erroneous. He further says, in Spain as elsewhere, the malady seldom, if ever, affects persons belonging to the middle or upper classes of society, but almost invariably attacks the poorly fed and badly lodged labouring population; particularly if living in dwellings which are damp, devoid of free ventilation, and whose occupants sleep on the ground floors, on in the same hovels with mules and donkeys, frequently wallowing among ordures, besides suffering from all the evils of indigence.

Besides these noxious causes specially adverted to by Dr. Webster, it would appear not only that innutritious diet acts powerfully, but even the kind of food which is consumed under such circumstances exerts a baneful influence. Thus, putrid fish, whereof residents near the sea-coast oftener eat than persons dwelling inland, to a certain extent, may explain the greater frequency of this malady in maritime districts, compared with central and more elevated regions. Again, not only deficiency of proper nutriment is injurious, but eating diseased hog's flesh, habitual intoxication produced by deleterious "aguardiente"—bad, fiery brandy—consumption of old, mouldy grain, a scanty supply of vegetables, the want of salt, and abstinence from animal food, prove equally detrimental.

After discussing one or two other important questions bearing upon the loathsome affection here brought under review, but which space prevents our further alluding to on the present occasion, the author subsequently observes, although no expectation can be reasonably entertained of curing confirmed leprosy by remedies, or even of arresting its fatal progress after a certain stage, still something may perhaps be accomplished towards alleviating the sufferings of those victims who ocsionally have become martyrs to such an inveterate and incurable disease.

Judging from the experience acquired at the Granada Leper Hospital it would seem through attention to diet, cleanliness, frequent bathing, and proper clothing, some alleviation of individual suffering may be occasionally accomplished; while the judicious employment of patients in manual occupations, according to their physical strength and individual capacity, by remaining much in open places, where refreshing, pure air may be breathed, aided by varied recreations, good effects are sometimes obtained. In short, the system pursued should mainly resemble that now followed at most well-regulated lunatic asylums. Of course local remedies must be also applied to ulcerated surfaces—usually soothing applications—while the medicines prescribed internally will comprise those chiefly of a tonic description. But in regard to anticipating any permanent beneficial result through medical treatment, such an expectation Dr. Webster considers as almost invariably nugatory. Speaking of the occasion when nearly fifty leprous inmates attended at chapel, during the performance of divine service, the author remarks rather feelingly that, at no public establishment of any kind ever previously inspected throughout Europe, had he witnessed such a disgusting sight as the spectacle these miserably afflicted fellow-mortals there presented to the eyes of professional or curious visitors. The disfigured, ghastly countenances of some, covered with deep, extensive sores: distorted features of others; the idiotic expression of many, and the feeble physical frames of most; at the same time that every person was then devoutly kneeling and engaged in prayer; formed altogether such a melancholy exhibition of frail humanity, that the scene baffled description, and therefore few persons would desire, it is believed, again to join in any similar ceremony, or one so lugubrious.

Finally, in concluding his notes respecting the Granada institution for lepers, of which we have now made a running commentary, in order so convey thereby some general notion of several salient points discussed in the present communication, Dr. Webster observes, according to numerous recognized facts contained therein, and which were all obtained from reliable authorities, that the following general inferences may be legitimately deduced:—

"1st. Leprosy chiefly affects the male sex, as it has always done heretofore.

"2nd. Every age is liable to its attacks, but mostly that after puberty and during manhood.

"3rd. The malady is not infectious, in the strict sense of that definition.

"4th. It seems to be an endemic disease.

"5th. Occurs only among the lower and badly fed ranks of society, at present.

"6th. Residents on the sea-coast constitute its ordinary victims in Spain, as elsewhere.

"7th. The principal apparent causes seem to be putrid, indigestible, and innutritious aliment; also indigence, filth, and occupying insalubrious dwellings in marshy, miasmatous soils; or where a humid, variable marine atmosphere prevails.

"8th. Leprosy may be communicated by inoculation,—according to some authorities.

"9th. It is incurable when fully developed; hence in the latter stages, all medical treatment proves unavailing.

"10th, and lastly. Although the disease seems nearly extinct in districts where leprosy formerly prevailed extensively, and has almost disappeared from every other European country excepting Greece and Norway, it yet still exists to some extent throughout Spain; especially among poverty-stricken natives, who live either upon or near the Southern Mediterranean sea-shores of that Peninsula."

During the middle ages, and subsequent to the crusades, leprosy was an exceedingly common affection even amongst the highest ranks of society. For instance, Don Alonzo, King of Arragon, died in 1284, of that malady, and King Robert the Bruce, of Scotland, also fell its victim after several years' sufferings. So common was leprosy throughout Great Britain, that not many centuries ago, upwards of one hundred lazar-houses existed in this country; indeed, the village of Libberton, near Edinburgh, derives its name from this disease being then very frequent, and hence originally called Lepper-town; while St. James's Palace at London, is said to have been first built as a receptacle for lepers. Now the complaint has almost wholly disappeared from Europe, with the exception of Spain and a few maritime regions where it still ravages the population; Greece, the sea-coast of Portugal, and Norway, being considerably devastated. In Iceland, likewise, according to Dr. Lindsay of Perth, leprosy exists to a considerable extent; and perhaps sporadically in other European districts. Therefore, its absence may be held as a sure indication of advanced civilization, in any country where so incurable and direful a disease proved at one period very rife and destructive among all classes of residents.

*Year-Book of Medicine and Surgery, and the Allied Sciences, for* 1860. Edited by Dr. G. Harley, Dr. Handfield Jones, Mr. Hulke, Dr. Graily Hewitt, and Dr. Sanderson, New Sydenham Society. London: 1861. 8vo, pp. 578.

This volume is a decided advance, both in arrangement and compilation, upon its predecessor, and it gives promise of a still better future. But why such a mis-begotten word as *Psychiatrik?*

*The Clinical Teaching of Psychology.* A Valedictory Address. By J. Crichton Browne, L.R.C.S. Ed., Senior President of the

Royal Medical Society of Edinburgh. Session 1860-61. Edinburgh, 1861. pp. 23.

An eloquent and instructive address from which we cull the following interesting paragraphs:—

"Once, indeed, in this country, long before the era of Pinel, clinical psychology promised fair to assume its proper position, for we find that one of the objects proposed at the foundation of St. Luke's, in 1751, was that 'of introducing more gentlemen of the faculty to the study and practice of one of the most important branches of physic.' The enlightened physician of this hospital at that time, Dr. William Beattie, tells us, too, in the preface to his 'Treatise on Madness,' that, 'by a unanimous vote the governors signified their intention of admitting young physicians, well recommended, to visit the hospital, and freely to observe the treatment of the patients confined.' But Dr. Beattie was far in advance of his age, and immediately after his death a retrograde movement in psychological matters took place. At this we cannot be surprised, when we remember that this was almost coeval with the time when Samuel Johnson, himself not free from the gloom of mental disease, and Foote, enlivened themselves with an occasional stroll among the lunatics of Bedlam, or with witnessing a hanging or judicial flogging; and when swarms of schoolboys paid twopence during the Easter holidays to see 'the fools' in the same hospital. This institution is said to have derived at one time an income of 400*l.* per annum from the exhibition of its inmates.

"Tracing the history of clinical psychology in England, we find no further effort at such tuition made for nearly a hundred years. In 1842, however, St. Luke's again opened its wards to students, and in the same year Dr. Conolly commenced lecturing at Hanwell. Dr. Sutherland and Dr. Hitchman have also lectured upon insanity; and now almost every English asylum receives a limited number of resident pupils. In Germany, clinical instruction in psychology appears to have originated with Horn, who lectured at Berlin in 1818. Horn was succeeded by Newmann, and Newmann by Ideler. Müller, Conradi, and Frank have also lectured on psychology in Germany, having illustrative insane cases introduced into their ordinary hospitals. Now, too, young medical men are admitted into some German asylums.

"At an early period in the history of reformed psychology, the celebrated Guislain gave lectures in Belgium which were afterwards collected and condensed into his valuable 'Leçons Orales;' whilst in Holland, Van der Kolk has strongly advocated the necessity for the study of mental disease.

"In France, perhaps more than in any other country, progress has been made in psychological teaching. Pinel having inaugurated a new *régime*, and introduced humane principles into asylum discipline, gave lectures on his favourite study, having as illustrations patients in Salpêtrière. This he did about 1814, and was followed in a few years by Esquirol, who lectured at the same hospital, and afterwards at the royal asylum of Charenton. M. Ferrus, M. Bottex, and M. Rech, have all been psychological teachers in France. M. Baillarger and M. Falret, both so well known in this country, have also contributed to the advance of psychological science by instructing in it, and France possesses many other distinguished lecturers upon insanity.

"In Scotland, the first lectures on the subject of insanity were those delivered by the venerable Sir Alexander Morrison, in 1827. They were so far clinical that they were illustrated by drawings of striking cases which had occurred in his own practice—a mode of teaching then a novelty. These pathognomical pictures furnished part of the drawings in Sir Alexander's large work.

"In 1836, Dr. W. A. F. Browne gave a course of five lectures at the Montrose

Asylum. They were sanctioned by the directors, but heard by a promiscuous audience, and were regarded as a bold step. They form the volume, *What Asylums were, are, and ought to be.* In 1840 we find the author of this work, in one of his annual reports, advocating systematic psychological teaching. We quote the passage, as it was the first shadowing forth of a principle which has since been widely adopted. After enumerating the high qualities necessary in a medical superintendent, he writes:—'Acting upon the conviction that such qualifications can rarely be found combined, and can only be acquired by long training and experience, it has been proposed to render the Institution a clinical school for the education of young medical men who propose to make insanity a special study. Under certain restrictions one or more students will be admitted as assistants to the physician, who will thus have the inestimable advantage of living among the insane, of watching and becoming acquainted with their habits of thinking and acting, and of performing every office which kindness dictates or treatment demands. For obvious reasons the whole of an asylum cannot be thrown open to all students indiscriminately, however desirable in some respects such an innovation might be; and these assistants are accordingly required to have received some instructions in medicine previous to their appointment, to be articled pupils of the superintendent, and to be members of the household.' In 1851, Dr. Browne again gave a course of lectures to his medical assistants at the Crichton Royal Institution, Dumfries, instructing them in the observation of mental disease, and teaching the application of the principles of philosophy and medicine in the modes of treatment now employed.

"In Edinburgh, for many years the absence of any systematic course of lectures on mental diseases constituted a great defect, but we have now two distinguished lecturers on the subject. Dr. Skae, the justly eminent superintendent of Morningside, commenced lecturing several years ago, and has since continued to give an annual summer course, which may be attended with much profit, not merely by students having Indian service in view, but by those entertaining other projects. It consists of systematic lectures on mental diseases, and visits to the asylum, where characteristic and striking cases are met and conversed with, and where the principles of asylum construction are demonstrated.

"Professor Laycock began giving a special summer course in 1859; but he had previously been in the habit of incorporating a series of lectures on insanity with his winter course on Practice of Physic. I would be lacking in diffidence were I to speak of Professor Laycock's ability for the task which he has imposed upon himself in so disinterested a manner. I will only remark, that his profound and extensive knowledge of philosophy, combined with his distinguished professional attainments and his experience, acquired whilst physician to an asylum in England, renders him peculiarly fitted to act as a teacher of psychology. The University of Edinburgh is deeply indebted to him for supplying what was an obvious defect in its medical department, by a course at once theoretical and practical.

"After all that has been said, we must surely be astonished that such 'a glaring anomaly should still exist as the exclusion from the prescribed course of study of one of the most important diseases in the whole range of nosology, and one which every practitioner must, sooner or later, meet in his every-day business.' * The consequences of this exclusion have been felt by some of the

* "In illustration of the growing and acknowledged necessity for incorporating special inquiry into mental diseases with general clinical instruction, I may mention that Professor Bennett, my distinguished teacher, since these words were delivered, has greatly enlarged his consideration of the subject in his clinical course."

most distinguished of our profession. The illustrious Dr. Alison, some years before his death, caused a psychologist to describe to him, at great length, the general paralysis of the insane, which he had never recognised; and very recently, gossip says that another psychologist was requested to read a monograph on the same subject, to a learned Society, the Medico-Chirurgical of this city, for the special behoof of its members. It is to be fervently hoped that a remedy for the evils which we have attempted to point out will be speedily applied. Some continental schools have, in this matter, set us an example which we would do well to follow by establishing regular clinical psychological courses. Perhaps a sanguine spirit may attribute to the unifaction and nationalisation of Italy, that a Psychological Clinique has been established in the University of Bologna, by a decree dated December, 1860."

*Suggestions concerning the construction of Asylums for the Insane.* Illustrated by a series of plans. By William Dean Fairless, M.D., &c., Resident Officer in charge of the Old Royal Lunatic Asylum of Montrose. Edinburgh, 1861. pp. 28.

We commend this pamphlet to the attention of all who are interested in the provision of Asylum accommodation for the insane.

*Statistical Report of Cases of Insanity treated in the Retreat, Castleton Lodge, near Leeds, and after its removal in June,* 1858, *at Mount Stead, near Ilkley, from Jan. 1st,* 1850, *to Dec.* 31, 1860. By G. Pyemont Smith, M.D. Edin., Resident Proprietor of Mount Stead, Lecturer on Medical Jurisprudence at the Leeds School of Medicine, &c.

Dr. Pritchard, of Abington Abbey, set the excellent example which in this Report is followed by Dr. Pyemont Smith. Dr. Smith has adopted the forms of tabulation made use of by Dr. Pritchard, "as more suitable," he writes, "than any other I could have substituted." We trust that other asylum proprietors will enter the course so ably pursued by these two gentlemen. But we would suggest the propriety of appending a summary of the most important results to any subsequent reports.

*The Errors of Homœopathy.* By Dr. Barr Meadows. London: 1861. pp. 45.—A vigorous exposure of this form of charlatanism.

We have also on our table a third edition of Dr. Brinton's admirable little work on the *Medical Selection of Lives for Assurance;* Dr. Halford's ingenious, but most brief observations *On the Time and Manner of Closure of the Auriculo-ventricular Valves;* the *Introduction to Swedenborg's Economy of the Animal Kingdom;* with address to the reader by Medicus Cantabrigiensis; the Rev. Edwin Sidney's *Second Visit to Earlswood* (June, 1861); Dr. Billod's *Relation d'une Visite à l'Asile des Idiots d'Earlswood, suivie de quelques Reflexions sur le no-restraint;* and of reprints, Mr. Dunn on *Medical Psychology;* Dr. Toulmin, *On the Importance of the Functions of the Skin in the Pathology and Treatment of Tubercular Consumption;* and Dr. Cormac's *Metanoia; a Plea for the Insane.*

